# Human Papillomavirus Type 18 *cis*-Elements Crucial for Segregation and Latency

**DOI:** 10.1371/journal.pone.0135770

**Published:** 2015-08-19

**Authors:** Mart Ustav, Fernando Rodriguez Castaneda, Tormi Reinson, Andres Männik, Mart Ustav

**Affiliations:** 1 University of Tartu, Institute of Technology, Tartu, Estonia; 2 Icosagen Cell Factory OÜ, Tartu, Estonia; 3 Estonian Academy of Sciences, Tallinn, Estonia; University of Nebraska-Lincoln, UNITED STATES

## Abstract

Stable maintenance replication is characteristic of the latency phase of HPV infection, during which the viral genomes are actively maintained as extrachromosomal genetic elements in infected proliferating basal keratinocytes. Active replication in the S-phase and segregation of the genome into daughter cells in mitosis are required for stable maintenance replication. Most of our knowledge about papillomavirus genome segregation has come from studies of bovine papillomavirus type 1 (BPV-1), which have demonstrated that the E2 protein cooperates with cellular trans-factors and that E2 binding sites act as *cis*-regulatory elements in the viral genome that are essential for the segregation process. However, the genomic organization of the regulatory region in HPVs, and the properties of the viral proteins are different from those of their BPV-1 counterparts. We have designed a segregation assay for HPV-18 and used it to demonstrate that the E2 protein performs segregation in combination with at least two E2 binding sites. The cooperative binding of the E2 protein to two E2 binding sites is a major determinant of HPV-18 genome segregation, as demonstrated by the change in spacing between adjacent binding sites #1 and #2 in the HPV-18 Upstream Regulatory Region (URR). Duplication or triplication of the natural 4 bp 5’-CGGG-3’ spacer between the E2 binding sites increased the cooperative binding of the E2 molecules as well as E2-dependent segregation. Removal of any spacing between these sites eliminated cooperative binding of the E2 protein and disabled segregation of the URR and HPV-18 genome. Transfer of these configurations of the E2 binding sites into viral genomes confirmed the role of the E2 protein and binding sites #1 and #2 in the segregation process. Additional analysis demonstrated that these sites also play an important role in the transcriptional regulation of viral gene expression from different HPV-18 promoters.

## Introduction

Human papillomaviruses (HPVs) infect the basal keratinocytes of the differentiating epithelium and induce benign tumors such as papillomas, warts and condylomas. These viruses are also considered the etiological agents responsible for anogenital and head and neck cancers [1] [2] [3]. Development of vaccines against HPV-6, -11, -16 and -18 has helped to prevent HPV-related diseases by protecting against infection. However, these vaccines have little effect on the millions of people already infected with one or more subtypes of HPV [4] [5]. An improved understanding of the mechanisms of replication and stable maintenance and segregation of viral genomes in infected cells will facilitate the identification of targets for drug-based interventions and the development of drugs to eliminate latent viral infections in patients.

Papillomaviruses (PVs) and herpesviruses (Epstein-Barr virus (EBV) and Kaposi’s sarcoma-associated human herpesvirus (KSHV)) maintain their genomes as multicopy nuclear extrachromosomal genetic elements in infected dividing cells [6] [7] [8]. The establishment of stable extrachromosomal replication of the PV genomes in infected proliferating basal/suprabasal cells is the result of two major functions: (i) viral DNA replication during the S and G2 phases of the cell cycle [9] [10] and (ii) segregation/partitioning of the viral genomes into daughter cells upon cell division during mitosis [11].

Papillomavirus DNA replication is initiated by the E1 and E2 proteins, which load the cellular replication machinery at the viral replication origin [12] [13]. In EBV and KSHV, the viral proteins EBNA1 and LANA1, respectively, initiate DNA replication during the latent infection phase [14] [15] [16]. In addition to initiating DNA replication, EBNA1 and LANA1 function in viral genome segregation to avoid the loss of copies of the extrachromosomal viral genome when the nuclear membrane breaks down during cell division. BPV-1, EBV and KSHV employ a similar basic mechanism in genome segregation in proliferating cells. The viral genomes are tethered to the mitotic chromosomes by a single viral protein: E2 for BPV-1, EBNA1 for EBV and LANA1 for KSHV [17] [18] [19]. These proteins bind to the mitotic chromatin receptors through viral protein-specific domains and can be detected by immunofluorescence (IF). IF-FISH analysis has revealed that these proteins co-localize with the viral genome on mitotic chromatin [11] [18] [20]. The proteins have similar organization comprising a C-terminal sequence-specific DNA binding/dimerization domains (DBD) that is responsible for binding at specific sites in the viral genome and an N-terminal multifunctional transactivation domain responsible for assembly of the segregation complex [21] [22] [23] [24] [25] [26]. The segregation complex comprises E2 and host factors that provide a physical link between the cellular chromosome and the viral episomal genome. The viral genomes that are tethered to the chromosomes are actively partitioned together with the cellular chromosomes into the newly forming daughter cell nuclei during cell division [11] [20] [19]. Most of our knowledge about the molecular mechanisms of papillomavirus segregation and the segregation complex has been obtained from studies of BPV-1. The BPV-1 E2 protein and at least six E2BS binding sites provide effective segregation of plasmids even without DNA replication, as measured by a specific segregation assay [27]. Full size and N-terminally truncated forms of the E2 protein can both function in the regulation of viral gene transcription and viral genome replication, but only the full-length dimeric E2 protein is capable of supporting segregation of the BPV-1 genome [28]. The N-terminal transactivation domain of BPV-1 E2 is responsible for interaction with the transcription machinery; it binds to the E1 protein and enables chromatin attachment. The partially overlapping but distinct functional structural domains in E2 are responsible for interaction with the transcriptional and replication machineries and for binding to the mitotic chromatin [11] [29] [30]. Separating the roles and impact of the different interactions in the formation of segregation complexes in the context of the viral genome is difficult. Analysis of the properties of chimeric and mutant E2 proteins have revealed that the segregation-competent complex contains multiple copies of the BPV-1 E2 protein, which bind to the chromatin and engage the cellular transcription complex in the segregation process. These components act in concert to ensure proper plasmid segregation [29]. A number of cellular proteins have been identified as putative components of the E2-dependent mitotic chromatin segregation complex, including the bromodomain protein Brd4 [31] [32] [33]. Brd4 forms a strong complex and co-localizes with BPV-1 E2 at the sites of BPV-1 chromatin attachment. Brd4 plays an important role in E2-dependent transcription activation [34] and a less pronounced role as the mitotic chromatin receptor for E2 [31, 34]. In addition to the interaction of E2 with Brd4, BPV-1 E2 co-localizes on chromatin with ChlR1, a DNA helicase that plays a role in sister chromatid cohesion. ChlR1 association is required for loading of the BPV-1 E2 protein onto mitotic chromosomes and represents a kinetochore-independent mechanism for viral genome maintenance and segregation. A ChlR1 binding-defective E2 mutant, W130R, binds Brd4 but is unable to maintain episomal genomes. [35]. E2 may also interact with the mitotic spindle [36] and with the mitotic kinesin-like proteins MKlp2 [37] and TopBP1 [38] during the viral genome segregation process. Thus, E2 may have many interactions with the mitotic receptor that are essential for tethering E2 and papillomavirus genome to mitotic chromosomes, as the segregation activity is provided by a segregation complex comprising E2 and several host factors.

The majority of human papillomavirus E2 proteins interact with Brd4, but Brd4 interacts with E2 protein during the regulation of transcription, hindering the study of viral replication and segregation [39–41]. E2 is localized at the chromatin-bound foci during different cell cycle phases. There are clear indications that other HPV proteins, such as E1 and E6, may have important roles in compartmentalization of E2 as well as in viral genome maintenance [42] [43]. Three types of E2-containing foci have been identified in human keratinocytes in interphase. The first type are authentic replication foci that form during S and G2 phases in human keratinocytes [44] and U2OS cells [9], which exhibit active ongoing viral genome replication and contain cellular and viral replication factors. These factors replicate the viral genome, while other factors are involved in DNA repair and recombination. Brd4 is localized adjacent to these foci [40]. The second type of foci are formed upon expression of the E1 and E2 proteins and transfection of an origin-containing replicon and do not require Brd4 for their formation. These foci are formed in the presence of E2 that is deficient for binding Brd4 [45]. The third type of foci contain E1, E2 and Brd4 but not replicon DNA. These data suggest that E1 can modulate the interaction of E2 with cellular factors, including Brd4 [40]. In C-33A cells [41], Brd4 is recruited to E1/E2 replication foci. Most of these studies have been performed using epitope-tagged E1 and E2 expression vectors that maintain efficient support of the viral origin of replication but do not necessarily reflect the actual expression levels of these proteins during normal viral infection. Overexpression of E1 induces a DNA damage response and growth arrest in these cells [44] [46] [9]. Thus, it has been difficult to study E2 mitotic chromatin binding in the presence of E1 protein in these experiments. Alpha-papillomavirus E2 proteins are not easily detected on mitotic chromosomes [47] and do not bind tightly to host chromatin with Brd4 [39]. Biomolecular fluorescence complementation has enabled the observation of the interaction of Brd4 with HPV16 E2 during all phases of mitosis [48]. This interaction is dependent on the phosphorylation of serine 243 in the hinge region of HPV16 E2 [49]. However, most of these data have been obtained in cell lines that do not support transient or stable replication of the viral genome or HPV-18 gene expression, and the relevance of these observations to HPV genome segregation remains unclear.

The interactions of HPV E2 with cellular proteins and with the viral genome are likely qualitatively and quantitatively different from the corresponding interactions of the BPV-1, EBV or KHSV segregation proteins. Attachment of the alpha-papillomavirus E2 proteins to either the mitotic chromatin or mitotic spindle has been observed, and these proteins can be detected on mitotic cell chromatin only in some phases of mitosis [36, 47]. The HPV8 E2 protein binds to specific regions of acrocentric chromosomes [50] [51]. However, the role of this E2 binding in the segregation process remains to be confirmed. The HPV genome and E2 protein have been detected in various foci in interphase cells, but the interactions of E2 with chromosomes or in other cellular compartments during different stages of mitosis are poorly understood. In addition, HPV genomes carry no more than four canonical E2 binding sites, in contrast to the 17 E2 binding sites of the BPV-1 genome, which may suggests a difference in the segregation process. Therefore, a critical question is whether studies on the segregation of the BPV-1 can be used to understand HPV segregation? There is a clear need to provide physical or functional proof that the HPV E2 protein and E2 binding sites are the major determinants of segregation of HPV genomes in mitotic cells.

Our objective of this study was therefore to clarify the roles of E2 and the E2 binding sites in segregation. In this study, we have developed a biological assay for measuring plasmid segregation based on the HPV-18 E2 protein and E2 binding sites, similar to the assay used for BPV-1 [11]. Using this segregation assay, we were able to demonstrate that two E2 binding sites, #1 and #2, can be considered as *cis*- elements for the segregation of the HPV-18 genome. These two E2 binding sites provide the platform on which the E2 protein binds in a cooperative manner and can form the segregation complex. Reducing E2 cooperative binding to these E2 binding sites reduces the segregation efficiency, and increasing E2 cooperative binding increases the segregation efficiency. Incorporation of these configurations of the E2 binding sites into the HPV-18 genome demonstrated that these two E2 binding sites constitute a platform for segregation complex assembly and segregation of the viral genome. Elimination of the cooperative binding of the two E2 dimers on these sites by removal of the spacer between E2BS #1 and #2 eliminates the segregation and stable replication of the HPV-18 genome. We also determined that, in the context of the genome, the segregation element plays an additional important role in modulation of viral gene expression by controlling the promoters that drive the expression of E1, E2 and other viral proteins. Our analysis demonstrates that the segregation element has additional important roles in transcription and replication during the establishment and maintenance of latent replication.

## Results

### Design and evaluation of the segregation assay for HPV-18

**Fig 1:**
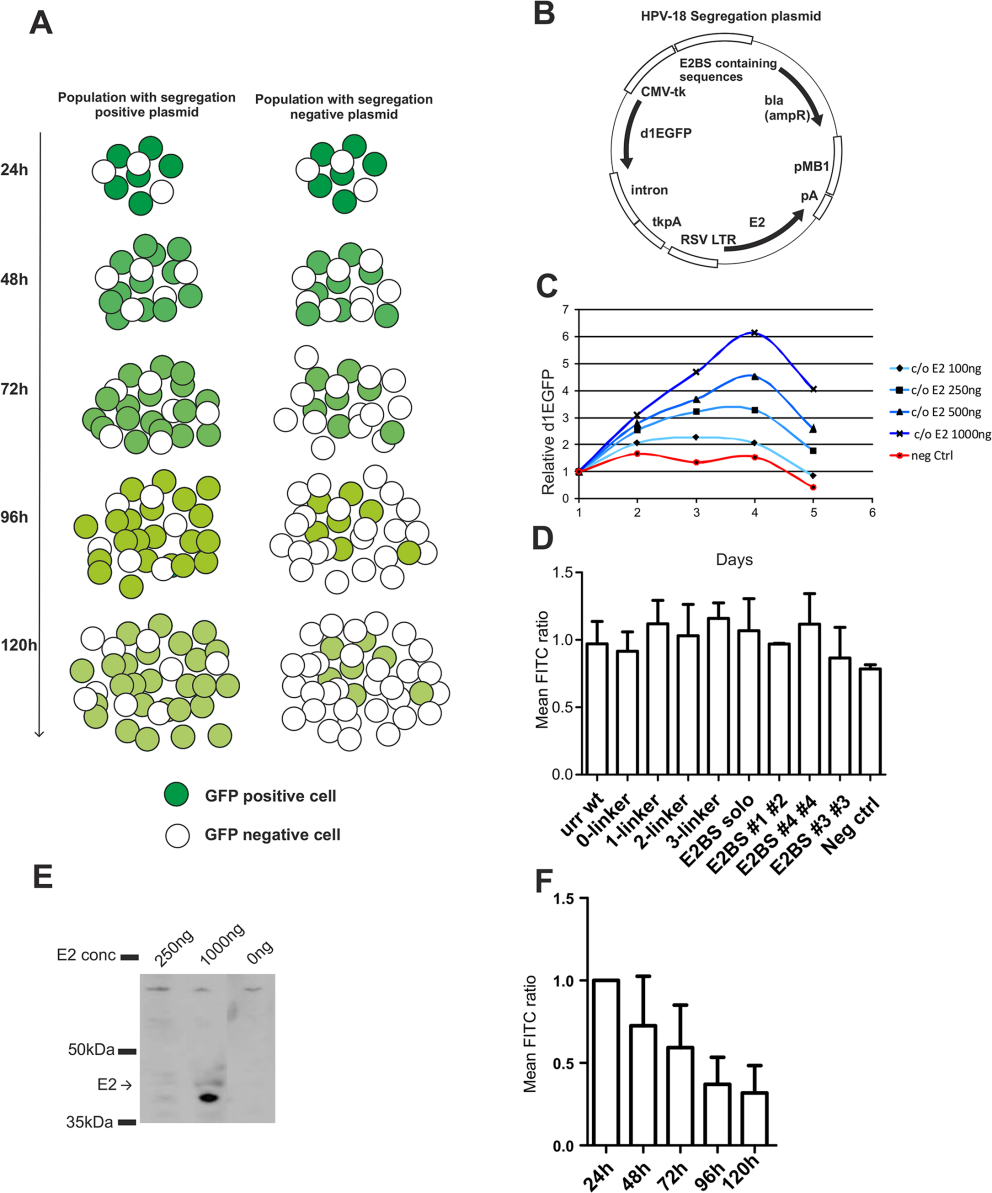
Overview of the segregation assay. **A** Illustration of the Jurkat cell-based segregation assay. In the segregation-positive GFP-containing population, an increase in GFP-positive cells over time can be observed. By contrast, the population lacking GFP plasmids with segregation properties shows no increase in GFP. **B** The HPV-18 segregation plasmid contains functional elements required for the segregation assay: (i) the cytomegalovirus (CMV) promoter for d1EGFP expression; (ii) the Rous sarcoma virus long terminal repeat (RSV-LTR)-driven HPV-18 E2 expression cassettes; (iii) a region for E2BS-containing sequences. **C** Partitioning assay of plasmids expressing HPV-18 E2 and HPV-18URR. A plasmid lacking the URR sequences was used as a reference for negative segregation. To assess the plasmid concentration dependence of the partitioning function, a series of input DNA dilutions was transfected. A clear correlation of input DNA concentration and partitioning efficiency is evident. **D** FITC mean values obtained from segregation assays that should reflect the effect of E2 and E2BS on d1EGFP expression. All segregation assays were performed with 1 μg of segregation plasmid unless otherwise indicated. **E** Western blot analysis of U2OS cells transfected with 250 ng and 1000 ng of segregation plasmid. Rabbit anti-HPV-18E2N antibody was used. **F** FITC mean fluorescence reduction of dividing cell populations transfected with the URR and E2 expression cassette containing segregation plasmid.

The functional segregation assay is based on a reporter plasmid containing an expression cassette for destabilized d1EGFP, an expression cassette for E2 and oligomerized E2 binding sites (E2BS) in the dividing cell population. This assay has previously revealed that segregation of the BPV-1 genome is dependent on chromatin attachment mediated by the viral E2 protein and its multiple binding sites [11]. The segregation assay is performed in a Jurkat cell line that is a suspension cell line and allows measuring d1EGFP expression from the same population of transfected cells and thereby allowing accurate calculations of cell division and changes of d1EGFP positive cells. We designed a similar assay for HPV-18. The segregation plasmid carried the following functional elements: (i) a cytomegalovirus (CMV) promoter-driven cassette for short-lived d1EGFP reporter expression; (ii) a Rous sarcoma virus LTR-driven expression cassette for HPV-18 E2; and (iii) the E2BS-containing sequences (HPV-18 URR region or different combinations of the HPV-18 E2 binding sites). A map of the plasmid is presented in Fig 1B. We analysed the effect of the E2 protein expression level on the segregation of the E2BS containing plasmids. We observed clear effect of the level of expression on this process. Therefore to analyse the segregation process in the Jurkat cells we selected the codon-optimized E2 cassette for our assays. The expression of E2 was analyzed in the normalized transfection assay using HPV-18 E2-specific antibodies (Fig 1E). The segregation/partitioning function of this plasmid is determined by measuring the change in the number of d1EGFP-positive cells during growth of the cells using FACS. As a result of an active segregation mechanism, the number of d1EGFP-positive cells increases due to segregation of the plasmid into daughter cells. The transfected plasmid copy number in cells decreases because the plasmid is degraded and diluted as a result of segregation during cell division. During cultivation, the number of plasmid-positive cells increases for all plasmids during the first timepoints as a result of movement of the transfected plasmid into the nucleus of the cell, where it becomes transcriptionally active. Eventually the transfection dependent expression reaches a plateau approximately 48h-72h post-transfection. At later time points, a reduction of the number of the plasmid positive cells is caused by the exhaustion of the pool of intracellular plasmid due to degradation and loss of the reporter plasmid (Fig 1A).

We analyzed the effect of different input concentrations of the segregation plasmids on segregation efficiency in Jurkat suspension cell culture. The number of d1EGFP-positive cells was measured by FACS analysis at different time points after transfection of 1 μg of the plasmid in the normalized experiment. As shown in Fig 1C, the plasmids expressing E2 exhibited an increase in d1EGFP-positive cells over time due to cell division, confirming functional segregation of the monitored plasmids. The segregation activity of the plasmid clearly correlated with the amount of transfected plasmid (Fig 1C). The negative control plasmid expressing E2 but lacking URR or E2BS sequences was transfected at the maximum concentration used for the titration of the segregation plasmids (1 μg). These data suggest that the HPV-18 URR plasmid segregation function is dependent on the E2 expression level and that removal of either of the viral *cis*-elements eliminates segregation of the plasmid. The role of the E2 protein in alpha-papillomavirus gene transcription is two-fold: it acts as a repressor by binding at the HPV early promoter proximal E2 sites and serves as a transcription activator at the early promoter distal E2BS [52]. In the normalized experiment, we evaluated the effect of E2 expression on d1EGFP production from the CMV promoter by analyzing d1EGFP fluorescence intensity 24 h after transfection. We observed that the d1EGFP expression level per cell, as indicated by mean fluorescence values, did not change considerably depending on the presence of the URR or other configurations of the E2 binding sites in the plasmid (Fig 1D). These data suggest that the HPV-18 URR plasmid segregation function is dependent on the E2 expression level and that removal of the viral *cis*-elements eliminates plasmid segregation.

### Segregation requires at least two E2BSs

**Fig 2:**
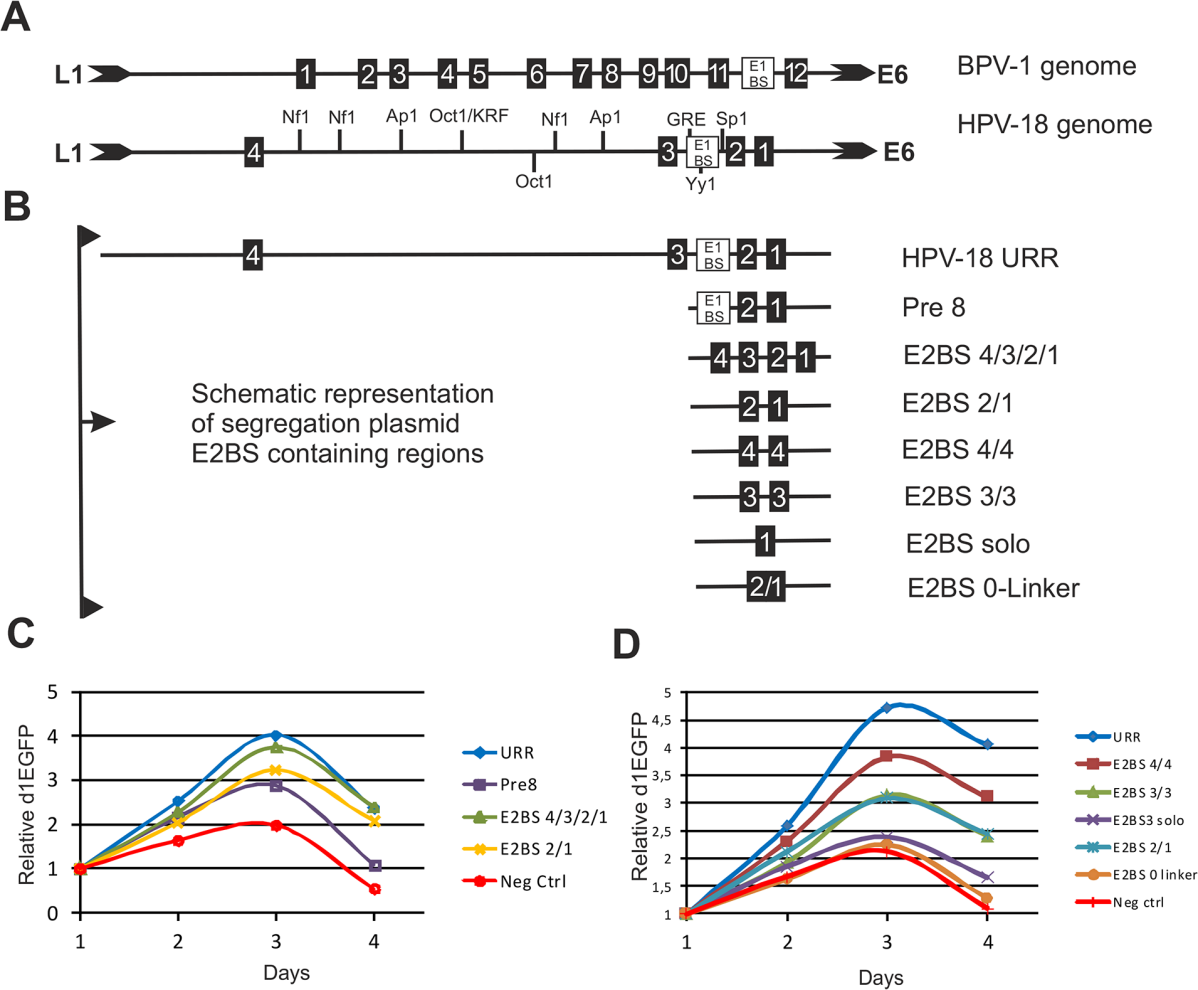
The HPV-18 segregation assay. **A** Schematic representation of the *Upstream Regulatory Region* (URR) of the BPV-1 genome and the HPV-18 genome. **B** Diagrams of the E2BS-containing sequences used in the segregation assay plasmids. **C** Partitioning assay with plasmids containing full-length HPV-18 URR, combinations of E2BSs separated by a four nucleotide (CGGG) linker and a truncated URR named Pre8 containing two E2BS sequences (nucleotides 7853–110). **D** Different pairs of E2BSs (E2BS #4, #3, #2 and #1) separated by the 4-nucleotide linker were used to estimate segregation efficiency. A single E2BS variant, E2BS3 solo, and E2BS with a 0-linker lacking the 4-nucleotide spacer, were used. Full-length HPV-18 URR was used as a positive reference for segregation. Two E2BSs were critical for effective segregation function. In addition, the linker sequence was crucial for effective E2 molecule binding to the plasmid. Plasmids lacking the linker sequence or containing a single E2BS did not segregate to daughter cells effectively.

The segregation assay demonstrated that E2 protein binding to its binding sites is absolutely essential for segregation. However, the HPV-18 URR contains many transcription factor binding sites in addition to the E2BSs. E2BSs are the only *cis-* elements required for the segregation/partitioning function of BPV-1, and thus we determined if this is also true for HPV-18. The setup and functionality of the HPV URR regions, including the number and location of the binding sites for E2 and cellular transcription factors, is different from that of BPV-1 and other HPV genotypes (Fig 2A). The BPV-1 URR carries 12 E2BSs, 6 of which are required for supporting viral genome attachment to the metaphase chromosomes and for segregation of BPV-1 plasmids to the daughter cells [17, 27]. By contrast, the majority of *alpha*-genus HPV URRs, including HPV-18, contain only 4 E2BSs (Fig 2A for HPV-18). To determine the importance of the E2BSs and the other transcription factor binding sites in the URR region for HPV-18 segregation and partitioning, a set of segregation plasmids was constructed carrying truncated URR sequences or E2BS sequences in different configurations (Fig 2B): 1) E2BS #3 and #4 as well as all sequences of other transcription factor binding sites in the region were deleted (Pre8); 2) the URR was replaced by all four HPV-18 E2BSs in tandem separated by the CGGG linker. 3) E2BSs #1 and# 2 were separated by the natural linker. In addition, plasmids were engineered with one E2BS site or two sites, either sites #4 and #3 or sites #1 and #2, with the spacer removed (Fig 2B).

Transfection of these plasmids into Jurkat cells revealed that the plasmids with truncated versions of URR—Pre8 carrying an E1 binding site as well as YY1, GRE and Sp1 binding sites and the plasmids with two E2BSs (#1 and #2) in their natural configuration without any additional transcription factor binding sites were as active in segregation as the plasmid with four E2BSs linked to each other with the CGGG linker sequence (Fig 2C). These data suggest that two E2BS sequences, #1 and #2, are sufficient for E2-dependent segregation of the plasmid.

Next we tested the effect of different E2BS combinations in the segregation assay. The sequences of E2BS #1 and #2 are identical, but E2BS #3 and #4 differ in the 4-nucleotide linker between the palindromes. These different E2BS sequences result in different E2 binding affinities [53]. We constructed segregation plasmids in which we duplicated the sequences of the identical E2BSs: E2BS #4/#4, E2BS #3/#3, E2BS #2/#1 (Fig 2B). The sites were linked with the same CGGG linker found between E2BS #1 and #2. E2BS #4/#4, which contained two copies of the highest affinity binding site, was more effective in the segregation assay than the E2BS #2/#1 and E2BS #3/#3 configurations. This result suggests that the E2 binding affinity for the two E2BS sequences is an important determinant of segregation. A plasmid containing a single copy of E2BS #1 (E2BS solo) was unable to segregate and was grouped with a negative control plasmid lacking E2BS. Interestingly, the segregation plasmid E2BS 0-Linker containing E2BS #1 and #2 without the 4 nucleotide CGGG linker was unable to support plasmid segregation and grouped with E2BS solo and the negative control plasmid (Fig 2D). Thus, we conclude that the HPV-18 segregation function is dependent on two E2BSs, with no absolutely critical role for other cellular *cis*-factor binding sites. These data suggest that at least two E2BSs are needed for the E2-dependent segregation activity of the plasmid.

### Cooperative binding of the two E2 dimers to the E2BSs is important for segregation

**Fig 3:**
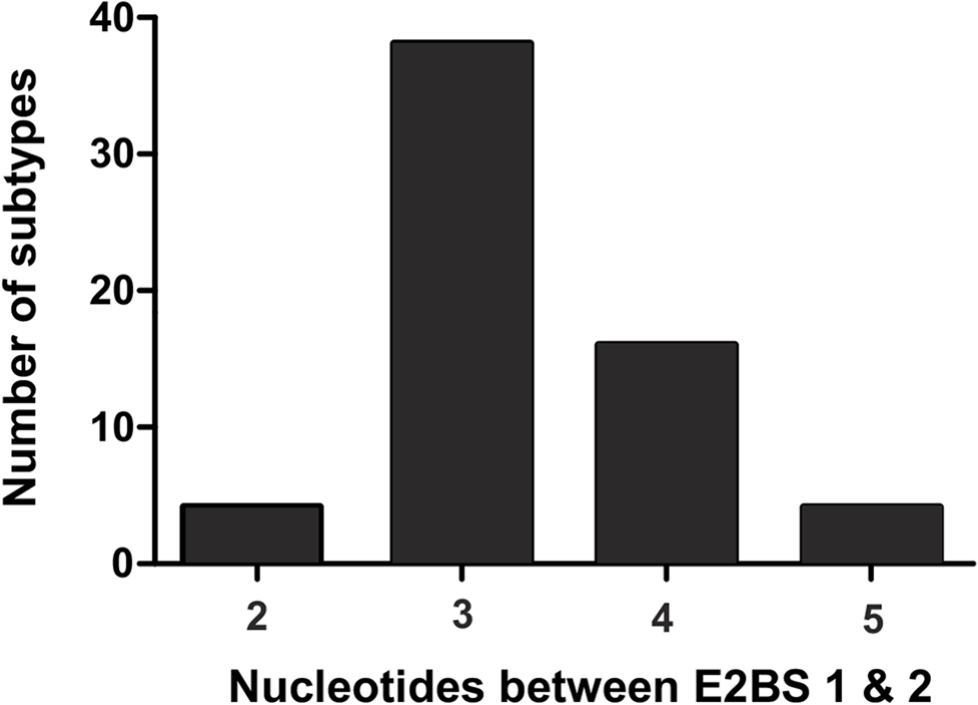
E2BS spacing among HPV’s. Distribution of nucleotide spacing between E2BS #1 and #2 of the 63 alpha genus papillomaviruses.

We determined if the CGGG linker between E2BS #1 and #2 is critical for segregation because removal of the spacer eliminated segregation in the segregation assay. The positioning of the E2BSs within the HPV genomes is somewhat conserved [54]. Alignment of the E2BS #1 and #2 regions of 64 *alpha*-genus papillomaviruses revealed that the spacing the two sites ranges from 2 to 5 nucleotides, with a 3 nucleotide spacing most prevalent (Fig 3). However, no conserved spacer sequence motif was identified.

**Fig 4:**
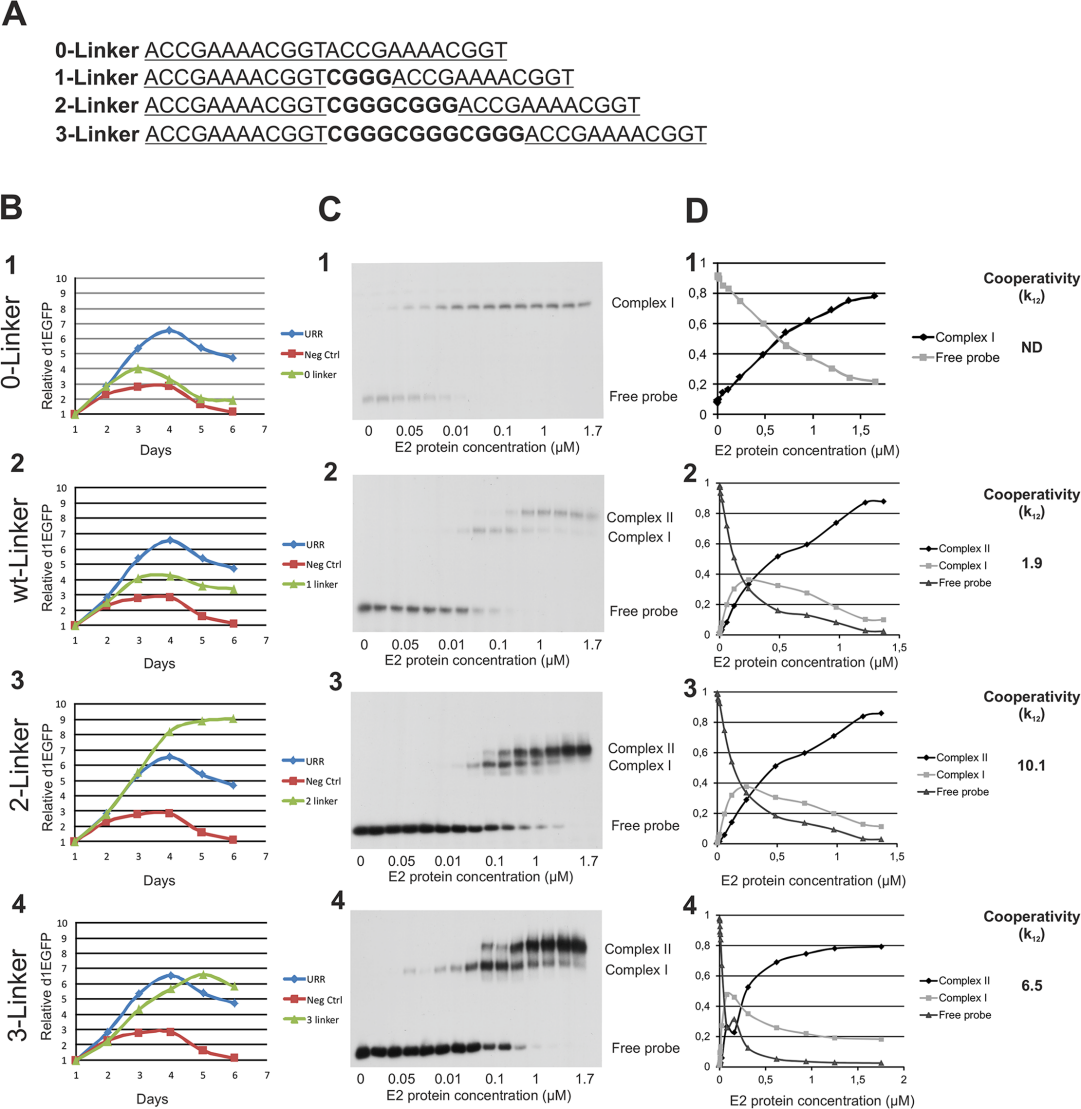
Plasmid segregation and link with cooperative binding of E2. Segregation assays and EMSAs of different E2BS probes. **A** Illustration of the E2BS #1 and #2 combinations used in the E2BS-containing region of the segregation assay plasmid and the γ-ATP labeled DNA probe sequence used for the EMSA. **B** Segregation assays of the 0-Linker, 1-Linker, 2-Linker and 3-Linker segregation plasmids. **C** EMSAs of the 0-Linker, 1- Linker, 2-Linker and 3-Linker probes using purified HPV-18 E2 protein. **D** Quantitative EMSA signals used to calculate cooperative binding factors for E2 protein to its binding sites. The fractional species abundances derived from the quantified bands in the gel are shown.

We constructed plasmids with zero, one or two copies of the spacer (Fig 4A) and analyzed the cooperative binding of the E2 dimers and segregation activity. We found that changing the spacing between E2BS #1 and #2 had a strong effect on both cooperative binding of E2 and segregation of the plasmid. We analyzed the effect of changing the spacing between the E2BSs on E2 cooperative binding using a gel-shift assay (Fig 4C and 4D). Baculovirus-expressed and purified E2 protein was used in the EMSA with four different dsDNA radiolabeled probes: dsDNA probes with E2BS #1 and E2BS #2 joined without a linker, joined with wild-type (wt) linker, or joined with 2- or 3-linker sequences (Fig 4A). The EMSA gel had 15 lanes. No E2 protein was added to the first sample, while the second to fifteenth samples contained increasing amounts of the baculovirus-expressed and affinity-purified HPV-18 E2 recombinant protein. The highest concentration of used HPV-18 E2 protein was 1.65 μM, which resulted in > 95% binding of the dsDNA radiolabeled probe, as determined by the quantitation of different migrating oligonucleotide species (Fig 4D). The EMSA results demonstrated that the 0-Linker sequence (Fig 4C1 and 4D1) was qualitatively different from the other three probes, indicating that two E2 dimeric proteins cannot bind simultaneously to the 0-linker oligonucleotide. This configuration of E2 protein binding sites eliminates the binding of the second E2 dimer to the oligonucleotide. The EMSA results for the other two modified probes (2-Linker and 3-Linker) (Fig 4C3 and 4C4) were similar to that for the wt probe and revealed three fractional species, indicating cooperative binding of the E2 proteins to the adjacent binding sites in all three configurations of the probes. The quantified blots and calculated cooperativity factors *k*_12_ are shown in Fig 4, panel D. The mean values for the cooperativity factor followed the order 1-Linker < 3-Linker < 2-Linker. Collectively, the data demonstrate a strong correlation between the positioning of the E2 binding sites and the cooperative binding of two E2 dimers to the DNA.

Removal of the linker between E2BS #1 and #2 eliminated plasmid segregation activity (Fig 2D and Fig 4B1). Duplication of the linker sequence, resulting in 8-nucleotide spacing, resulted in a considerable increase in segregation efficiency that exceeded even that of the plasmid with the entire URR region with 4 E2BSs (Fig 4B3). Three copies of the linker (12 nucleotide spacing) resulted in segregation efficacy similar to that of the URR-containing plasmid (Fig 4B4). These results suggest that E2BS spacing plays an important role in the cooperative binding of E2 dimers to DNA and contributes to PV genome segregation in the context of E2BSs #1 and #2. These data suggest that the different segregation efficiencies provided by the 0-, 1-(wt), 2- and 3-linker constructs may result from differences in cooperativity in binding to the E2 sites. Collectively, the data indicate a strong correlation between segregation efficiency and cooperative binding of E2 to the DNA. However, it is surprising that HPVs do not use the most efficient configuration of E2 binding sites for segregation. Another important activity in the HPV-18 genome may be dependent on this *cis*-sequence.

### E2BS #1 and #2 spacing is crucial for HPV-18 genome stable replication

**Fig 5:**
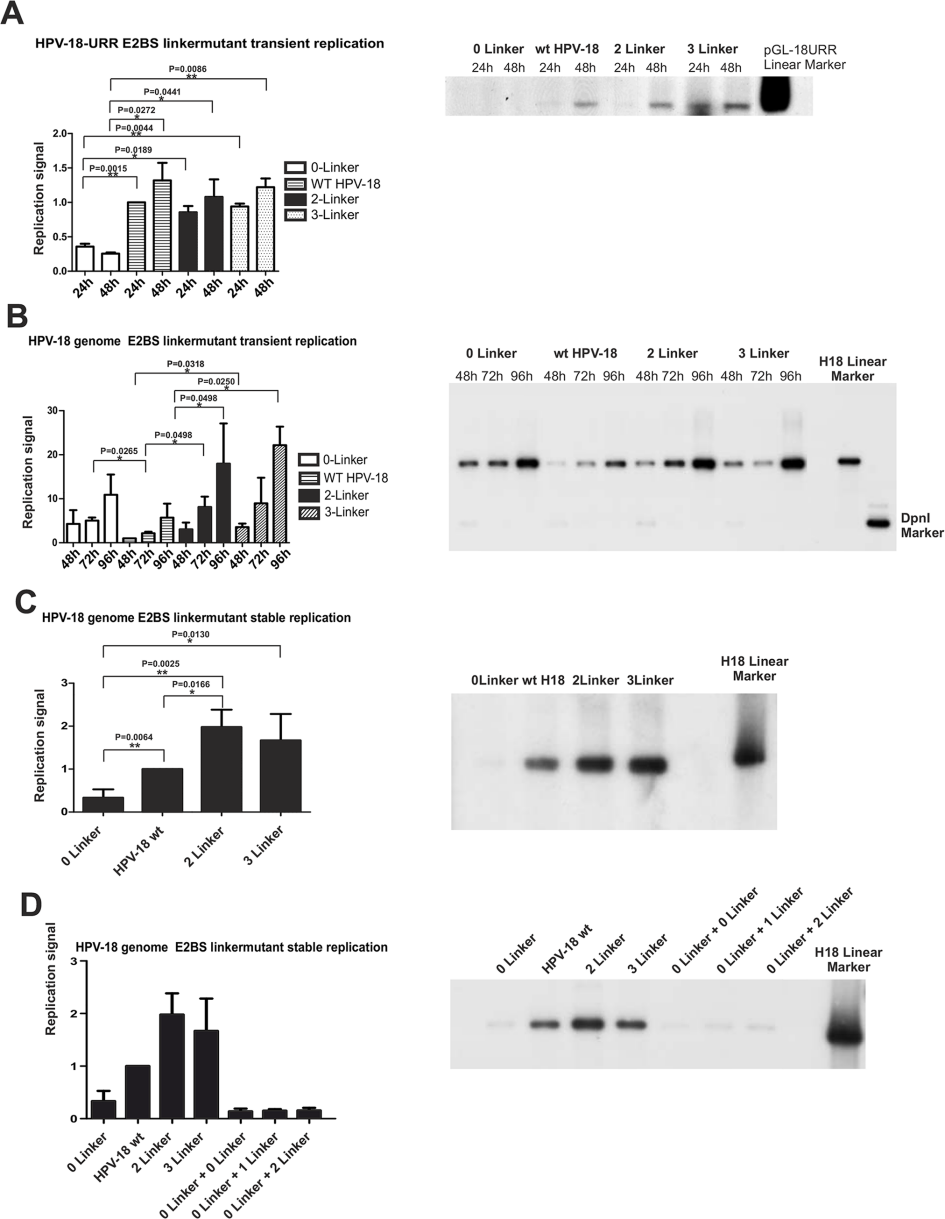
HPV Replication assays. **A** Transient replication assay of HPV-18 URR plasmid E2BS #1 and #2 linker sequence mutants (0-Linker, 1-Linker (wt), 2-Linker, 3-Linker). 25 ng of the E1 and E2 expression vectors were cotransfected, and total DNA extracts were obtained 24 h and 48 h post-transfection. DNA was linearized using Hind III enzyme, and input DNA was eliminated by digestion with DpnI. Normalized qPCR data are presented in the left panel, and the Southern blot analysis is shown in the right panel. A 2-sample t-test was performed and statistically significant (P<0.05) samples are indicated **B** Transient replication assay of the HPV-18 E2BS #1 and #2 linker sequence mutant genomes (0-Linker, 1-Linker (wt), 2-Linker, 3-Linker). Total DNA extracts were obtained 48 h, 72 h, and 96 h post-transfection. DNA was linearized using BglI, and input DNA was eliminated by digestion with DpnI. Normalized qPCR data are presented in the left panel, and the Southern blot analysis is presented in the right. Linker modification results in upregulated replication intensity compared to the wt genome (1 Linker). A paired t-test was performed and statistically significant (P<0.05) samples are indicated **C** Stable replication assay of HPV-18 E2BS linker sequence mutant genomes. HPV-18 genomes were cotransfected with pBabePuro in U2OS cells and maintained under puromycin selection for 12 days, after which total DNA extracts were obtained. DNA was linearized with BglI. Normalized qPCR data are presented in the left panel, and the Southern blot analysis is presented in the right panel. A paired t-test was performed and statistically significant (P<0.05) samples are indicated **D** Additional E2 binding site #1 and #2 configurations were introduced at the BbsI site of the HPV-18 0-Linker genome, and a stable replication assay was performed. Normalized qPCR data are presented in the left panel, and the Southern blot analysis is presented in the right panel.

We next evaluated the importance of the spacer between the E2BSs in papillomavirus DNA replication. The E2BS #1 and #2 spacer region was mutated in the context of the URR in the pGL-18URR plasmid and in the HPV-18 genome by changing the wt sequence to the 0-,2- and 3-linker configurations (Fig 4A). For the replication studies of the URR plasmid and the HPV-18 genomes, the human osteosarcoma cell line U2OS, which is a suitable model system for studies of transient and stable replication of the papillomavirus genome [55] [9] [56] [57], was used. First, we conducted the E1 and E2 protein-based replication assays using 100 ng of the pGL-18URR plasmids co-transfected with 25 ng of E1 and E2 expression vectors to measure the replication efficiency 24 and 48 h post-transfection for the wt and mutated origins. As shown in Fig 5A, the removal of the linker sequence between E2BS #1 and #2 resulted in a 5-fold decrease in replication efficiency evaluated by qPCR. This result is consistent with the EMSA results, which demonstrated that the E2 protein binds to the mutant version as a single dimer, to either E2BS #1 or #2, but not to both sites simultaneously. Therefore, this configuration of the E2BSs is less efficient at loading the E1 protein onto the replication origin. Thus, sites 1 and 2 are the major factors controlling initiation of replication from the HPV-18 origin, and other sites (#3 and #4) do not compensate for the defect in replication. The addition of linker sequences between the E2BSs (2 or 3 linkers) seems to result in a a slight decrease of the ability to initiate DNA replication compared to the wt configuration (Fig 5A).

In contrast to the URR plasmid transient replication assay, in which E1 and E2 were provided from the separate expression vectors, the transient replication signals of the mutant HPV-18 minicircle genomes analyzed at 48, 72 and 96-h timepoints indicated increased replication efficiency compared to wt HPV-18 (Fig 5B). All viral genomes carrying a modified linker region between the E2BSs, including the 0-linker variant, replicated at substantially higher (approx. 5 fold) levels than the wt HPV-18 genome in the transient replication assay measured up to 96 h post-transfection. These data suggest that, in addition to the role of E2BSs in replication and segregation, E2BS #1 and #2 are clearly important for the regulation of replication factor expression (E1 and E2) or the expression of transcriptional repressors. The difference between the effect on replication of the URR and the intact viral genome suggests that the spacing between E2BS #1 & #2 plays an additional role beyond its function in replication and segregation that may include transcriptional regulation of replication proteins. The effect of the 0-linker configuration is even more pronounced when the reduced replication efficiency in the E1 & E2-dependent replication of the URR is considered. E2BSs located immediately upstream from the viral promoter P102 clearly modulate its E2 protein-dependent function as a *cis*-repressor sequence [58]. Thus, the 0-Linker mutation causes a decrease in the cooperative binding of E2 and may cause unbalanced expression of viral replication and/or regulatory proteins that results in increased expression of replication proteins and therefore more efficient transient replication, regardless of the suppression of the initiation of replication from the URR by five-fold. To analyze the effect of these linker modifications on the stable replication of the HPV-18 genome, transfected cells were selected using the co-transfected linear selection marker plasmid pBabePuro. The growth medium was substituted with a puromycin-containing medium 72 h post-transfection. The cells were passaged in puromycin-containing medium for 4 days to remove untransfected cells. Then, the selective medium was replaced with regular growth medium, and the cells were grown under subconfluent conditions and regularly passaged every third day up to 14 days. Total DNA was extracted on day 15 and analyzed by Southern blot and qPCR. In contrast to the efficient amplification during the transient DNA replication, the copy number of the 0-Linker mutant genome decreased to nearly undetectable levels (Fig 5C). The 2-Linker and 3-Linker plasmids exhibited increased viral copy numbers compared to the wt genome, as observed in the transient replication assay. Thus, the potency of the stable replication and maintenance (measured as relative copy number of the viral genome) is correlated with segregation ability, demonstrating that increased cooperative binding of E2 to its binding sites results in enhanced segregation and partitioning of the genome. We also attempted to demonstrate that the defect in segregation introduced by removal of the linker sequence could be compensated by insertion of the normal E2BS configuration. We inserted extra E2BSs (0-Linker, wt-Linker and 2-Linker spaced E2BS #1 and #2) into the HPV-18 0-Linker mutant genome at the BbsI site located in the L1 ORF in an attempt to complement the impaired segregation function of the 0-Linker genome. However, as shown in Fig 5D by qPCR and Southern blot, this complementation did not rescue the segregation function of the 0-linker construct, indicating that insertion of these binding sites considerably reduced replication of the viral genome in stable (Fig 5D) as well as transient replication assays (data not shown). There is no clear explanation for the observed changes in replication efficiency for the L1 linker insertions. However, a disruption in effective transcription of viral genes may be responsible by introducing the E2BSs to the L1 ORF. Therefore, we conclude that the natural positioning of E2BSs #1 and #2 is crucial for their concerted functions in transcription, replication and segregation of the viral genome.

### E2BS #1 and #2 spacing affects HPV-18 transcription

**Fig 6:**
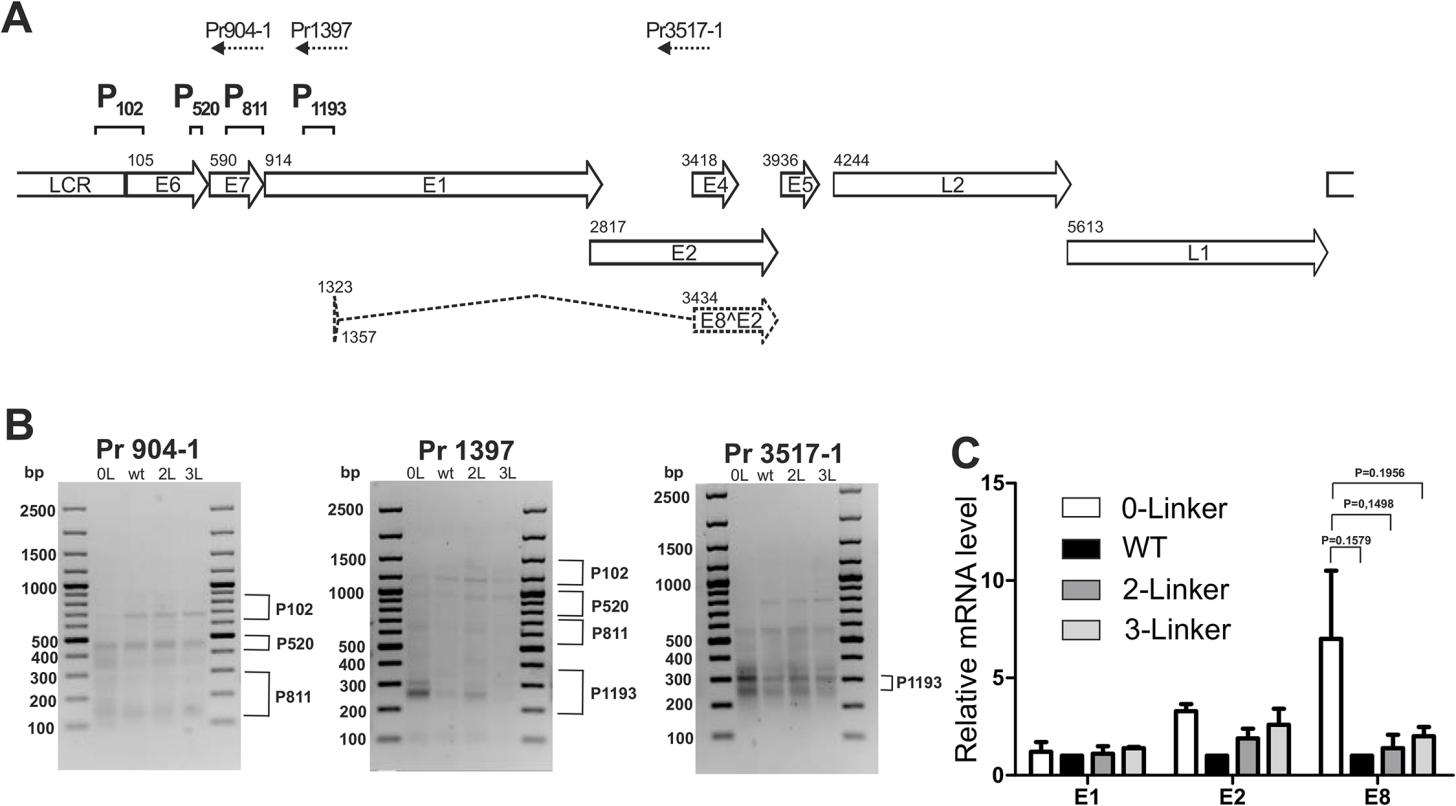
5’RACE and mRNA analysis. 5’ RACE assay. **A** HPV-18 genome and primers used in the 5’RACE assay to detect HPV-18 TSS. **B** 5' RACE analysis of RNAs in U2OS cells with an HPV-18-specific primer (Pr904-1) that binds to a site in the E7 ORF, an HPV-18-specific primer (Pr1397-1) that binds to a site in the E1 ORF and an HPV-18-specific primer (Pr3517-1) that binds to a site in the E4 ORF. Products representing the RNAs initiated from promoters P_102_, P_520_, P_811_ and P_1193_ are indicated. **C** Relative mRNA levels of E1, E2 and E8^E2C from the 0-Linker, wt HPV-18, 2-Linker and 3-Linker genomes. A paired t-test was performed and obtained P values are indicated.

We previously defined the HPV-18 transcription map in U2OS cells during transient replication, stable maintenance and vegetative amplification by identifying viral promoter regions, transcription polyadenylation and splicing sites during HPV-18 genome replication. Mapping of the HPV-18 transcription start sites in U2OS cells revealed five distinct promoter regions (P_102_, P_520_, P_811_, P_1193_ and P_3000_). [57]. Using the HPV-18 specific primers Pr904-1, Pr1397 and Pr3517-1 ([57]; Fig 5A) the transcripts initiated from each promoter can be distinguished in a 5’RACE assay. This method allows a comparison of the expression levels of viral genes and the viral replication negative regulator E8^E2C [28] [57] because the protein is primarily encoded by the promoter P_1193_-initiated transcripts. To determine whether the changes in spacing between E2BSs #1 and #2 have an effect on the promoter activities of HPV-18, we performed 5’-RACE analysis of the mutant genomes with 0, 2 and 3-Linkers between E2BS #1 and #2 and compared the results with those for the wt genome. Fig 6B shows the relative changes in promoter usage for the mutant genomes. Compared to the wt genome, the 0-linker mutation results in a significant decrease in transcripts initiated from P102 (analysis with Pr904-1 in Fig 5B). P_102_ is the main viral promoter and controls the expression of nearly all viral early proteins, with the exception of E8^E2C. P_102_ is the only promoter responsible for the expression of replication factor E1. Another significant change in relative promoter activity was observed for the E8^E2C promoter P_1193_. The activity of P_1193_ was significantly increased in the context of the 0-linker mutation (Pr904-1 and Pr3517-1 in Fig 5B). To quantify the levels of different transcripts, we performed qPCR using cDNA prepared from the 5’RACE assay reflecting the relative levels of transcripts encoding E1, E2 and E8^E2C. As shown in Fig 6C, there was an increase in E2 transcripts for all mutant genomes, particularly for the 0-Linker genome, which might also explain the improved replication efficiency. Interestingly, an increase in E8^E2C was also observed, but instead of transcriptional repression, we observed activation of viral transcription, which indicates that E8^E2C may act as part of a negative regulatory feedback loop and that binding of E8^E2C is compromised, particularly in the context of the 0-Linker mutant genome. Thus, the effects of the linker substitution mutants on the replication and maintenance of the viral genome evidently result from changing the relative balance of the levels of viral transcripts, including those encoding viral replication factors and repressor proteins. These data suggest that the natural configuration of the E2BSs has been evolutionarily established as optimal for the regulation of the transcription, replication and segregation of the HPV-18 genome.

## Discussion

HPV genomes must encode two major functions to persist in proliferating cells during latent viral infection. First, the viral genomes must be able to replicate during S and G2 phases prior to each cell division [9] [10]. Second, the viral episomes must segregate nearly evenly into the newly formed nuclei during cell divison to avoid viral genome loss in some cells and excessive accumulation (jack-pot cells) in others. Both stages of stable maintenance must be functional, regulated and controlled to prevent the loss of viral genome copies.

The E2 protein is a key mediator of effective episome segregation into daughter cells in BPV-1. BPV-1 has 17 E2BSs, of which 12 are located in the URR. We have demonstrated that at least 6 E2BS sites are required for effective episome segregation [27]. The HPV-18 genome contains only four E2BSs located in the LCR that could be involved in viral genome segregation/partitioning. Therefore, we sought to analyze the role of the HPV-18 E2 protein and the E2BSs in the segregation process. We designed and validated a segregation assay for HPV-18 with the objective of understanding whether the E2 protein and E2BSs have the same central role in segregation demonstrated for BPV-1. The transient segregation assay of HPV-18 allowed us to monitor plasmid persistence and loss kinetics without replication for up to 7 days and without engagement of the E2 protein or E2BSs in the replication or transcription processes, which would be complicated when the viral genome was used. The segregation assay demonstrated that E2 is the major viral factor and that E2BS #1 and #2 are essential *cis*-elements for the segregation of the plasmid during cell division. This *cis*-element functions in an E2 protein-dependent manner. Elevation of the E2 level increases the efficiency of plasmid segregation. The relative positioning of these sites determines the cooperativity of E2 binding to the DNA and also determines the efficiency of segregation.

The segregation assay revealed that the segregation efficiency is determined solely by the E2BSs and that the transcription factor binding sites in the URR do not play an apparent role in segregation. The sequences of E2BSs are not identical and can differ in the internal sequences of the 6 nucleotides in the binding site, and some E2BSs have lower affinity for E2 than others [53]. To determine whether there are any differences in segregation due to E2 binding to different binding sites, we conducted segregation assays with different combinations of E2BSs, as shown in Fig 2D. We observed that E2 binding to different sites correlated with the findings that HPV-18 E2 has a higher binding affinity for two copies of E2BS #4. Two copies of E2BS #3 and the paired sites #1 and #2 resulted in similar plasmid segregation properties. Plasmids with only one E2BS or plasmids lacking the CGGG linker between the two E2BSs did not exhibit segregation and acted much like the segregation plasmids used as negative controls. E2BS locations are structurally conserved in the URR of the alpha genus papillomaviruses [54]. Alignment of the regions containing E2BS #1 and #2 from 64 alpha genus papillomaviruses revealed that the spacing between these sites ranged from 2 to 5 nucleotides, with 3- and 4-nucleotide spacers most prevalent (Fig 3). We analyzed the effect of changing the positioning of the E2BS sequences relative to each other and evaluated different copy numbers of the CGGG spacer between the sites. Duplication of the linker resulted in alignment of the two E2BSs on the same steric side of the DNA strand, whereas three copies of the linker resulted in two sites on different sides of the DNA strand. The duplication of the linker region enhances segregation dramatically, exceeding even the efficiency achieved with the full–length URR. The plasmid with three copies of the linker also exhibited higher efficiency of segregation matching that of the full-length URR-containing plasmid. This result led us to form a hypothesis about the role of cooperative binding of E2 to the E2BS sequences and the correlation of binding affinity with segregation efficiency. Cooperative binding of E2 to its binding sites has been demonstrated previously but has not been attributed to any specific function [59] [60]. We demonstrated that changing the cooperativity of E2 binding by modulation of the spacer length between E2BS #1 and #2 has an effect on segregation. Cooperative binding of E2 was studied by EMSA. As shown in Fig 4C, one E2 protein was unable to occupy both sites simultaneously in the 0-linker construct. The wt 4-nucleotide linker configuration exhibited cooperative binding with a cooperativity factor of 1.9 (Fig 4D2).The 2 and 3-linker sequence configurations exhibited increases in cooperative binding of E2 protein of 10.1- and 6.5-fold, respectively (Fig 4D3 and 4D4). The cooperative binding of E2 to its binding sites correlates with the segregation efficiency of the plasmid. Therefore, cooperative binding of E2 determines the efficiency of segregation. These data allowed us to conclude that two E2 dimers bound to the DNA in a cooperative fashion are capable of assembling the complex with the host factors on the DNA, which mediates the segregation of the plasmid. The configurations of E2BS #1 and #2 are largely conserved, and we conclude that this is a *cis*-regulatory element for segregation of alpha-papillomaviruses genomes.

The wt URR and the URRs with two or three copies of the linker between the E2 binding sites replicated in the transient assay at more-or-less similar rates, whereas the URR with the 0-linker replicated at a 5-fold lower level. These data suggest that increased cooperative binding of E2 to these sites does not have an effect on E1 loading and the initiation of replication, and the binding of only one E2 dimer on the E2 binding site severely compromised the replication efficiency of the plasmid. Thus, other E2BSs do not appear to contribute much to the initiation of replication. Transferring the same configurations of E2 sites into the HPV-18 genome context and analyzing transient replication of the mutated genomes in U2OS cells yielded a surprising result. All HPV-18 genomes with a modified linker between the E2BSs exhibited increased activity in transient replication compared to the wt genome. High replication efficiency was particularly unexpected for the 0-linker sequence. This result strongly suggests that the natural configuration of E2 binding sites mediates the feedback control of gene expression and that changes in spacing between the E2BSs change the transcriptional efficiency by disrupting transcriptional repression control. In the stable replication assay shown in Fig 5C, the 0-Linker HPV-18 minicircle genome, which effectively replicated in the transient assay, lost most of the HPV-18 genome copies after the cells were passaged for 14 days (Fig 4C). This result is in agreement with data obtained from the segregation assay, which demonstrated that two E2BSs are required for segregation and that the 4-nucleotide spacer between these sites is crucial for effective segregation. Additionally, the segregation efficiency and viral genome copy number in the stable replication assay correlate with the cooperative binding efficiency, which indicates that higher cooperative binding of E2 to its sites results in enhanced segregation and higher viral copy number.

We have recently published a study in which we present the full transcription map of the HPV-18 genome replicating in U2OS cells and demonstrate that it is essentially identical to the transcription map of HPV-18 in keratinocytes [57]. The HPV genomes with altered linker sequences all exhibited increased replication efficiency compared to the wt HPV-18 genome, indicating that the modifications must have affected the activity of early gene promoters. To determine how the linker changes altered the promoter activity, a 5’RACE assay was performed using HPV-specific primers. For the 0-Linker, the HPV-18 genome exhibited a clear increase in transcription from P_1193_ (Pr3517-1 in Fig 6B). P1193 is the promoter used for production of E8^E2C, a repressor of viral replication [28] [57]. Although we observed an increase in E8^E2C repressor transcripts, we did not detect any repression of transcripts for the 0-Linker genome. However, we observed increased replication levels even though E1 and E2-dependent replication initiation of URR were compromised. This is an indirect result of E8^E2C repression due to binding at E2BS #1 and #2, which is required, and E8^E2C binding to the genome, which forms a negative regulatory feedback loop.

It has been suggested that E2 binding sites mediate different functions in the high-risk HPV URR [52]. The role of mediating E2 repressor function has been attributed to E2BS #1 and #2, whereas E2BS #3 and #4 have been assigned to E2-dependent transcriptional activation [61]. These sites are also targeted for differential methylation of their CpG motifs, and it has been suggested that hypermethylation may modulate viral transcription and replication. When they are proximal to early promoters, sites #1 and #2 are thought to mediate E2-dependent transcriptional repression and E1-dependent replication. Hypermethylation of these promoter-proximal sites could activate transcription of viral early genes [62]. Our data are in agreement with these E2BS methylation studies and confirm that full-length E2 protein and its truncated versions regulate gene expression through E2 binding to sites #1 and #2.

## Materials and Methods

### Plasmids and primers

The HPV-18 segregation plasmid is illustrated in Fig 1B. Generation of the minicircle HPV-18 genome has been described previously [63]. The E2BS #1 and #2 linker mutations were introduced into these genomes. Details of the cloning and plasmid sequences will be provided upon request. For the replication assays, the plasmids pMHE1-18 and pQMNE2-18 for HPV-18 E1 and E2 expression were used; these plasmids have been previously described [64]. pGL-HPV-18URR has been previously described [28]. The 0-Linker, 2-Linker and 3-Linker mutations were also introduced into this plasmid to construct pGL-HPV-18URR-0 Linker, pGL-HPV-18URR-2 Linker and pGL-HPV-18URR–3-Linker.

### Cell lines and transfections

For the segregation assay, Jurkat cells were grown in Iscove’s modified Dulbecco’s medium (IMDM) supplemented with 10% fetal calf serum (FCS). Transfections were performed via electroporation (210 V, 1000 μF) using a Bio-Rad Gene Pulser II with a capacitance extender (Bio-Rad Laboratories). U2OS cells were obtained from American Type Culture Collection (ATCC no: HTB-96), and grown in Iscove’s modified Dulbecco’s medium (IMDM) supplemented with 10% FCS. U2OS cells were transfected with the indicated amounts of HPV-18 miniplasmid by electroporation (220 V, 975 μF) using a Bio-Rad Gene Pulser II with a capacitance extender (Bio-Rad Laboratories).

### Antibodies

HPV-18 E2N antibodies were raised by immunizing rabbits with a truncated version of the HPV-18 E2 protein (amino acids 2–208). Approximatly five month old New Zealand rabbits (4) were initially immunized by 2 subscapular injections (into both side of body). The protein antigen was administred 4 times in amount of 0.1 mg in complete Freund adjuvant (only first immunization) or in incomplete Freund adjuvant, respectively. Finally the response was boosted by intravenous injection of 0.1 mg protein antigen. Suffering of animals was alleviated by ketamine/xylasine and euthanasia was performed by exsanguination under the anesthesia. The polyclonal antibodies were affinity purified on columns on which E2N protein had been immobilized. All animal work was conducted under FELASA guidelines.

### Baculovirus expression and HPV-18 E2 purification

Baculovirus expressing the HPV-18 E2 protein with an N-terminal strep-II tag was constructed using the Invitrogen Bac-to-Bac system according to the manufacturer’s instructions. The pFastBac-strpIIE2HPV recombination vector used to generate E2-expressing baculovirus was constructed by inserting the codon-optimized HPV-18 E2 cDNA from pCG-18E2c/o [28] between the XbaI and XhoI sites of pFastBac1-strepII (a gift from Ivar Ilves, University of Tartu).

The high-titer baculovirus stock was used to infect SF9 insect cells grown as a monolayer in 15-cm tissue culture dishes at a density of 10^7^ cells per dish. The cells were washed from the dishes 48 h after infection the by pipetting up and down and transferred to 15-ml falcon tubes for harvesting via centrifugation at 500 x g for 5 min. The medium was removed by suction, and the insect cell pellet was preserved at -80°C before cell lysis and protein purification. A total of 7 (15-cm diameter) tissue culture dishes of HPV-18 E2 baculovirus-infected cells were used for protein purification. The cell pellet was lysed in 1 ml of lysis buffer (20 mM Tris-HCl [pH 8.0], 30 mM KCl, 400 mM NaCl, 0.1 mM EDTA, 10 mM dithiothreitol [DTT], 0.5% (v/v) Tween-20, 5% (v/v) glycerol, 1x protease inhibitor mix [Roche] and 1 mM PMSF) for 10 min on ice and homogenized with a dounce homogenizer using 10 strokes of a B piston. The HPV-18 E2 lysate was cleared by centrifugation (14,800 rpm in a table-top minicentrifuge at 4°C for 20 min) and loaded onto a 1-ml strep-tactin sepharose column (IBA-lifesciences). The column was then washed 5 times with 1 ml of washing buffer (100 mM Tris-HCl [pH 8.0], 400 mM NaCl, 1 mM EDTA), and the streptavidin-HPV-18 E2 recombinant protein was eluted in 6 fractions of 0.5 ml of elution buffer (100 mM Tris-HCl [pH 8.0], 400 mM NaCl, 1 mM EDTA and 2.5 mM desthiobiotin).

### Segregation assay

The segregation assay was performed as described [11] [29]. Briefly, 3x10^6 cells were transfected in 250 μl of IMDM media supplemented with 10% FCS. The transfected cells were washed with 5 ml of the same media and were plated on 60-mm cell culture dishes in 5 ml of IMDM medium with 10% FCS. Samples for flow cytometry analysis of the number of d1EGFP-positive cells were withdrawn at 24-h intervals post transfection. The cell suspension volume was maintained at 5 ml throughout the assay, and the cells were diluted accordingly to maintain optimal density in the culture. Total cells and the GFP-positive cell population were quantified, and the plasmid segregation kinetics were calculated as described [11]

### Electrophoretic mobility shift assay (EMSA)

EMSAs were performed similar to a previously described method [29]. ^32^P-Labeled double-stranded DNA probes were prepared by incubating 100 ng of the single-stranded oligonucleotides with 5 μl of [γ^32^-P]-ATP and 10 U of T4 polynucleotide kinase for 30 min at 37°C. The reaction was stopped by adding 1 μl of 0.5 M EDTA (pH 8.0), and 100 ng of the complement strand oligonucleotide was added. The mixture was heated at 85°C for 5 min and allowed to cool to 55°C for completion of the annealing reaction. The labeled dsDNA was ethanol precipitated and washed with 70% (v/v) cold ethanol. The DNA pellet was dried and resuspended in 20 μl of TE buffer. The sequences used to prepare the E2BS probes are shown in Fig 4A. The reactions for the EMSA were assembled in a total volume of 5 μl. The reactions contained 1 μl of 5X EMSA buffer (20 mM HEPES [pH 8.0], 2.5 mM KCl, 1 mM DTT, 10% [v/v] glycerol, 0.1% [v/v] Nonidet P-40); 5 μg of polydIdC; 5 ng of sonicated salmon sperm DNA; 6.25 μg of BSA and 2.25 μl of mock lysate for the sample loaded in the first lane. The amount of purified HPV-18 E2 protein varied from 9.8 to 1.65 μg. Finally, 0.1 ng of the radioactive E2 binding site dsDNA probe was added to the mixture and incubated at room temperature for 20 min before loading into a [0.25 x Tris-Borate-EDTA] 6% polyacrylamide gel (80:1 acrylamide-bisacrylamide). The gel had been pre-run at 120 V for 20 min. Electrophoresis was performed for 90 min at 120 V. The gel was vacuum dried and then exposed to a phosphoscreen for 45 min. The screen was scanned using a Typhoon phosphorimager (GE Healthcare), and the different lanes were analyzed with Image Quant TL software (GE Healthcare). In each lane, three bands were quantified: the free dsDNA probe, which had the highest mobility; the first E2-shifted DNA band (with intermediate mobility); and the second E2-DNA complex, which had the lowest mobility. For the DNA probe without a linker, no intermediate band was observed due to the non-cooperative nature of the probe. The quantified bands were summed, and each band was treated as a fraction of the total sum. The analysis of the fractions was performed with the aid of the SimFit program (Manchester University). The cooperativity constant (*k*_12_) was obtained from a global analysis of the non-linear fit of the three species fractions to the following three equations, as performed by Seanar and Brenowitz [65].

1. Θ_0_ = 1/*Z*
2. Θ1 = (*k*_1_ + *k*_2_)*L/*Z*
3. Θ2 = (*k*_1_ *k*_2_ *k*_12_)*L ^2^/Z

Z is the binding polynomial, which is equal to 1 + (*k*_1_ + *k*_2_) * L + (*k*_1_ *k*_2_ *k*_12_)*L_2_. The constants *k*_1_ and *k*_2_ are the microscopic equilibrium association constants for intrinsic binding sites 1 and 2; *k*_12_ is the cooperativity constant that applies when both sites are bound. These microscopic equilibrium constants can be replaced by the two macroscopic equilibrium constants defined by *K*_1_ = *k*_1_ + *k*_2_ and *K*_2_ = *k*_1_ *k*_2_ *k*_12_. *K*_1_ and *K*_2_ can be determined from a single mobility-shift experiment.

For HPV-18 E2 binding to the dsDNA probes consisting of E2 binding sites -1 and -2 joined by various linker sequences, it is assumed that the two binding sites have identical affinity for the ligand. This assumption stems is based on the identical nucleotide sequences of E2 binding sites #1 and #2 and allows the cooperativity to be determined exactly. For this specific case, the data can be analyzed directly using the microscopic binding and cooperativity constants *k*_*i*_ and *k*_12_ because *K*_1_ = 2*k*_*i*_ and *K*_2_ = *k*_*i*_^*2*^ *k*_12_. This analysis was conducted for the one, two and three-linker dsDNA probes. In addition, the binding isotherm of the second E2 protein-DNA complex was used to derive the apparent affinity constant *k*m by fitting the hyperbolic curve with the SimFit program.

### DNA replication analysis

DNA from transfected U2OS cells was extracted using total DNA lysis. For the E1 and E2 expression vector-based transient replication assay, 24 and 48-h timepoints were used for DNA lysis. For the HPV-18 minicircle transient replication assay, 48, 72 and 96-h timepoints were obtained. The stable replication assay was performed as follows: U2OS cells were co-transfected with HPV-18 minicircle genomes and linearized pBabePuro vector. At 72 h post-transfection, the IMDM+10% FCS medium was substituted with IMDM+10% FCS medium containing 2 μg/ml puromycin. The cells were cultured under these conditions until the untransfected cells were dead (approximately 2–3 days); the medium was then replaced with IMDM+10%FCS. Cells were cultivated until they reached semiconfluency, after which semiconfluency was maintained until 14 days post-transfection. Then, the cells were lysed to isolate the total DNA. All DNA extracts were purified by phenol-chloroform extraction and ethanol precipitation. The purified DNA was digested with an appropriate enzyme for linearization and DpnI to remove input plasmid, resolved on an 0.8% agarose gel, blotted, and hybridized with an HPV-18 specific probe for the expression vector-based transient replication assay and the HPV-18 genome sequence-specific probe for minicircle replications that were labeled with [-32P]dCTP using random priming (DecaLabel kit; Fermentas). Specific HPV replication signals were detected by autoradiography exposure of X-ray film (Fuji).

### HPV-18 qPCR

The viral genome copy number in the U2OS cells during replication was analyzed by quantitative real-time PCR (qPCR). For each qPCR reaction, 10 ng of total DNA from the transient and stable replication assays was used, and the reactions were performed with EvaGreen qPCR Mix Rox (Solis BioDyne) according to the manufacturer’s protocol on a 7900 HT Fast Real-Time PCR System (Applied Biosystems). The HPV-18 genome replication signal was amplified with the following oligonucleotides (300 nM each per reaction): 5’-GCGCTTTGAGGATCCAAC-3’ (HPV-18 nt 110–127) and 5’-GTTCCGTGCACAGATCAG-3’ (HPV-18 nt 148–165, complement strand). For the pGL-18 URR plasmids, the following oligonucleotides were used: 5’-CATCTTACGGATGGCATGAC-3’ and 5’-CAACGATCAAGGCGAGTTAC-3’. The analysis was performed using the comparative threshold cycle (ΔCt) method. The results were calculated from the PCR cycle number in which the HPV signal exceeded the threshold value (Ct_HPV_). As a normalization standard, Ct_GAPDH_ was determined for the GAPDH gene sequence in the U2OS genome using the following oligonucleotides (300 nM each): 5’-TACTAGCGGTTTTACGGGCG-3’ and 5’-ACAGGAGGAGCAGAGAGCGA-3’. The relative value C_N_, which reflects the average viral genome copy number per cell, was calculated from the data using the formulas ΔCt = Ct_HPV_—Ct_GAPDH_ and C_N_ = 2^-ΔCt^.

### RNA extraction, rapid amplification of cDNA ends (RACE) and qPCR

PolyA^+^ RNA templates were extracted from U2OS cells that had been transfected with 1 μg of the HPV-18 genome minicircle by Dynabeads mRNA DIRECT Kit (Life Technologies). Then, 500 ng of polyA^+^ RNA was used as a template for 5’ RACE. 5’ RACE assays were performed with the SMARTer RACE cDNA Amplification Kit (Clontech) according to the manufacturer's instructions. The positions of the HPV-18-specific primers that were used for the amplification of the RACE products are indicated in Fig 6A; the sequences of these primers have been described previously [57].Products were separated on a 1.5% TAE agarose gel. The cDNA prepared by 5’RACE was used to evaluate the level of viral transcripts by qPCR. The primers and sequences used to detect E1, E2 have been published [55], and E8^E2C levels were detected using the following oligonucleotides: E8 Fwd 5’-TGGCTGTTCTGAAGTGGAAG-3’ and E8 Rev 5’-GGTGCTGGAATACGGTGAG-3’.
